# *MtSIN1a* Enhances Salinity Tolerance in *Medicago truncatula* and Alfalfa

**DOI:** 10.3390/genes16101156

**Published:** 2025-09-29

**Authors:** Huanyu Yue, Yuxue Zhang, Yafei Liu, Feng Yuan, Chuanen Zhou, Yang Zhao

**Affiliations:** 1 School of Life Sciences, Shandong University, Qingdao 266237, China; 15949852359@163.com (H.Y.); zhangyuxue2018@163.com (Y.Z.); liuyafei1995082018@163.com (Y.L.); czhou@sdu.edu.cn (C.Z.); 2 Shandong Key Laboratory of Precision Molecular Crop Design and Breeding, Qingdao 266237, China; 3 The Key Laboratory of Plant Development and Environmental Adaptation Biology, Ministry of Education, Qingdao 266237, China; 4 Inner Mongolia Pratacultural Technology Innovation Center Co., Ltd., Hohhot 010000, China; mengcaoyf666@163.com

**Keywords:** salinity tolerance, alfalfa, *MtSIN1a*

## Abstract

**Background/Objectives**: Alfalfa is a widely cultivated high-quality forage crop, and salinity tolerance is one of the most important breeding goals. *Glycine max SALT INDUCED NAC 1* (*GmSIN1*) was found to enhance salinity tolerance in soybean plants. The phylogenetic analysis showed there were two homologs of *GmSIN1* in *Medicago truncatula*, *MtSIN1a* and *MtSIN1b*. This raised questions regarding the roles of *MtSIN1s* in alfalfa under salinity stress. **Methods**: From a *Tnt1* mutant collection, we identified the mutants of *MtSIN1a*. We recorded the survival rate and plant height of *mtsin1a-1* and *mtsin1a-2* after 100 mM NaCl treatment. Subsequently, we generated *35S:MtSIN1a-GFP* transgenic alfalfa lines via genetic transformation. Two lines with relatively high *MtSIN1a* expression, *35S:MtSIN1a-GFP*#3 and *35S:MtSIN1a-GFP*#4, were selected for gradient NaCl treatments. In addition, DAB and NBT staining were performed, and the H_2_O_2_ content and catalase (CAT) activity were determined. Then, we used RNA-seq analysis and RT-qPCR to study the mechanism of its tolerance. **Results**: This study found that after salt treatment, the survival rate and plant height of *mtsin1a-1* and *mtsin1a-2* were significantly lower than those of the WT. The mutants of *MtSIN1a* were sensitive to salinity stress. The transgenic alfalfa plants exhibited higher plant height, weaker DAB staining, stronger NBT staining, less H_2_O_2_ content, and enhanced CAT activity. The transgenic alfalfa constructed by transforming *MtSIN1a* showed enhanced salinity tolerance with elevated ROS scavenging. We identified *MsSOD1* showing elevated expression levels in transcriptomic analysis. **Conclusions**: *MtSIN1a* is a positive regulator for enhancing salinity tolerance in alfalfa with activated ROS scavenging.

## 1. Introduction

Soil salinization severely inhibits plant growth and reduces crop yields, and is a major factor leading to global land degradation and hindering sustainable agricultural development [1,2]. The total area of salinity-affected land is 954 million hectares worldwide, posing significant threats to agricultural production and ecological sustainability [3]. Consequently, enhancing the productivity and sustainability of salinity-affected land represents a global challenge [4,5,6].

Currently, over 60 types of transcription factors (TFs) have been identified in plants. Among these, NAC (NAM, ATAF1,2, and CUC2), AP2/ERF (APETALA2/ethylene responsive factor), bHLH (basic helix-loop-helix), bZIP (basic leucine zipper), WRKY, and MYB transcription factors are closely associated with abiotic stress responses [7]. The NAC transcription factor family represents a class of plant-specific regulatory proteins that play crucial roles in diverse biological processes, including plant growth, development, and stress responses [8,9]. Soybean *GmSIN1* promotes ABA and Reactive Oxygen Species (ROS) accumulation by binding to the promoters of *GmNCED3s* and *GmRbohBs*, effectively amplifying the initial salt stress signal and enhancing salinity tolerance [10].

Salt stress impairs plant growth and development, as follows. (1) Osmotic stress: High concentrations of soluble salts elevate the osmotic pressure in the soil surrounding the plant roots. This hyperosmotic environment lowers the water potential at the root surface, reducing the plant’s capacity to absorb water. (2) Ion toxicity: Salt stress leads to an excessive accumulation of Na^+^ within plant tissues. This disrupts the Na^+^/K^+^ balance, resulting in the inhibition of enzymes involved in primary metabolism, glycolysis, and other pathways, ultimately causing damage to plant growth [11]. (3) Oxidative stress damage from ROS accumulation: While low concentrations of ROS can act as primary signaling molecules enabling plants to better respond to stress conditions, their excessive accumulation under salt stress causes oxidative damage. ROS primarily include hydrogen peroxide (H_2_O_2_), superoxide anion (O_2_^−^), and singlet oxygen (^1^O_2_). Respiratory burst oxidase homolog (Rboh) genes encode plasma-membrane-localized NADPH oxidase proteins, which serve as key enzymatic sources of apoplastic ROS [12]. Excessively accumulated ROS under salt stress cause damage such as lipid peroxidation in cell membranes, DNA damage, and protein denaturation. Plants mitigate the over-accumulation of ROS by activating ROS-scavenging mechanisms. The plant ROS-scavenging system primarily comprises two categories: enzymatic antioxidants and non-enzymatic antioxidant compounds. Key enzymatic antioxidants include superoxide dismutase (SOD), catalase (CAT), glutathione peroxidase (GPX), and ascorbate peroxidase (APX). Major non-enzymatic antioxidants include reduced glutathione (GSH), ascorbic acid (vitamin C), and phenolic compounds [13].

Alfalfa (*Medicago sativa*) is a perennial leguminous forage with high nutritional quality, yield potential, good palatability, extensive root architecture, and robust nitrogen-fixation capacity, establishing it as a globally important forage [14,15,16]. Characterized as a moderately salt-tolerant species, alfalfa exhibits strong ameliorating capability for soil salinization [17]. However, high salt stress severely restricts alfalfa growth, resulting in substantial yield reduction [18]. Alfalfa is an allotetraploid with cross-pollination behavior and a complex genetic composition, making direct genetic improvement challenging. The diploid *M. truncatula*, a close relative of alfalfa, serves as a model species for alfalfa research due to its small genome, short life cycle, and high genetic transformation efficiency [19].

In this study, we characterized two homologous genes of soybean *GmSIN1* in *M. truncatula*, namely *MtSIN1a* and *MtSIN1b*. *MtSIN1a*’s knock-out mutants displayed a salt-sensitive phenotype, and the *35S:MtSIN1a-GFP* transgenic alfalfa showed enhanced salt tolerance. Overexpressing *MtSIN1a* led to a significant reduction in H_2_O_2_ content and a significant increase in SOD expression levels and CAT enzyme activity, thereby enhancing alfalfa’s tolerance to salt stress. This work demonstrates a positive role of *MtSIN1a* in regulating salt stress response in Medicago, and therefore presents a novel way to breed for salt-tolerant alfalfa in the future.

## 2. Materials and Methods

### 2.1. Plant Materials, Growth Conditions, and Salt Treatment

In this study, *M. truncatula* ecotype R108 and *M. sativa* (alfalfa) SY4D were used as the wild type. The *mtsin1a-1* (NF14784), *mtsin1a-2* (NF10957) mutants were obtained from an *M. truncatula Tnt1* retrotransposon insertion mutant collection [20]. Seeds were extracted from mature pods, dried for two weeks, scarified with sandpaper, and placed on moist filter paper for germination at 4 °C for 7 days. When the roots reached 1 cm in length, the seedlings were transplanted into moist soil in a plant growth incubator under controlled conditions: a long-day photoperiod (16 h light/8 h dark), with temperature maintained at 21–23 °C, relative humidity at 60–70%, and light intensity at 150 μmol m^−2^ s^−1^. For alfalfa vegetative propagation, stem cuttings were taken from healthy mother plants before flowering. Stems were cut approximately 5 cm below the apex, and the basal ends were trimmed at an angle to maximize the cutting surface area. All expanded leaves were removed, and the ends were briefly soaked in water and then dipped in rooting powder (Anhui Wuwei County Flower Fertilizer Factory, Wuhu, Anhui, China) before being gently inserted into moist, loose soil. Stem cuttings were maintained in an incubator under the same growth conditions described above. Root initiation was observed after approximately two weeks.

Salt treatment: WT and mutant plants of *M. truncatula* were grown in plant growth incubator. Uniformly grown seedlings were transplanted into pots. Three seedlings were planted per pot. Plants of the same genotype were placed in the same tray. After acclimatizing in the plant growth chamber for three weeks, plants at the same vegetative growth stage were subjected to salt stress via irrigation with 100 mM NaCl solution [21]. The NaCl solution was poured into the tray approximately twice a week for four consecutive weeks. The survival rates were calculated in the end of the assay.

Stem cuttings of *M. sativa* SY4D and the transgenic lines *35S:MtSIN1a-GFP* were rooted in the plant growth incubator for about two weeks before being transplanted into individual square pots. One seedling was planted per pot. Plants of the same genotype were placed in the same tray. After acclimatizing in the plant growth chamber for four weeks, well developed plants were selected for salt treatment. A gradient of NaCl concentrations was applied incrementally, stepping from 100 mM to 150 mM, 200 mM, and finally 250 mM [22].

### 2.2. Phylogenetic Analysis

To identify the homologs of GmSIN1, we used the amino acid sequences of GmSIN1 for a BLASTP search in *Arabidopsis thaliana*, *M. truncatula* (https://phytozome-next.jgi.doe.gov/, accessed on 15 August 2025), and *M. sativa* (https://modms.lzu.edu.cn/, accessed on 15 August 2025). Twenty homologous sequences from *M. truncatula*, twenty homologous sequences from *A. thaliana*, and forty-eight homologous sequences from *M. sativa* with query cover ≥ 50% and identity ≥ 50% were used for the analysis (Supplementary Table S1). To study the phylogenetic relationships, the protein sequences were aligned in the ClustalW program (https://www.genome.jp/tools-bin/clustalw, accessed on 15 August 2025), and a neighbor-joining phylogenic tree was constructed with 1000 bootstrap replicates in MEGA11 [21].

### 2.3. DAB Staining

The DAB staining solution (1 mg/mL) was prepared by dissolving DAB powder in distilled water, followed by pH adjustment to 5.8 using NaOH. To protect it from light, the solution was wrapped in aluminum foil and stored at 4 °C until use. Healthy and intact leaves were collected and fully immersed in the DAB solution. Samples were incubated in the dark at 28 °C for 8 h in a constant-temperature incubator. After incubation, the DAB solution was discarded, and leaves were transferred to 75% ethanol for destaining. Chlorophyll was removed by boiling the samples in a water bath with repeated ethanol changes until the leaves became completely decolorized. Due to increased tissue fragility after destaining, samples were preserved in 100% ethanol to maintain their structural integrity before observation and imaging [23].

### 2.4. NBT Staining

The NBT staining solution was prepared by dissolving NBT powder in phosphate buffer (pH 7.8) to a final concentration of 0.5 mg/mL. The solution was protected from light with aluminum foil and stored at 4 °C for short-term use. Healthy and fully expanded leaves were selected and completely immersed in the NBT solution. Samples were incubated in the dark at 28 °C for 3 h in a constant-temperature incubator. After incubation, the NBT solution was discarded, and leaves were transferred to 75% ethanol. Chlorophyll was removed by boiling the samples in a water bath with repeated ethanol changes until complete decolorization was achieved [23].

### 2.5. Determination of Malondialdehyde (MDA) Content

The malondialdehyde (MDA) content, an indicator of lipid peroxidation, was measured according to the thiobarbituric acid (TBA) method. The absorbance of the supernatant was measured at 450 nm, 532 nm, and 600 nm [24]. We calculated the MDA content per gram of fresh weight using the following formulas:

MDA concentration (μmol/L) = 6.45 × (A_532_ − A_600_) − 0.56 × A_450_

MDA content (μmol/g) = MDA concentration (μmol/L) × extract volume (mL)/fresh weight (g)/1000

### 2.6. Determination of H_2_O_2_ Content

Fresh plant leaf samples were rapidly frozen in liquid nitrogen immediately after collection and were ground directly. The hydrogen peroxide content was then determined using the Beyotime Hydrogen Peroxide Assay Kit (Beyotime, Shanghai, China) following the manufacturer’s instructions.

### 2.7. Catalase Activity Assay

Fresh plant leaf samples were immediately frozen in liquid nitrogen after collection and were ground directly. CAT enzyme activity was determined using the Beyotime Catalase Assay Kit (Beyotime, Shanghai, China) following the manufacturer’s standard protocols.

### 2.8. PCR, RT-PCR Analysis

Using genomic DNA from the WT (*M. truncatula* ecotype R108), *mtsin1a-1*, and *mtsin1a-2* as templates, an 839-bp fragment of the *MtSIN1a* genomic sequence was amplified by PCR with primers MtSIN1a-F1 (5′-ATTCAAGACAAAGACCCACTTGC-3′) and MtSIN1a-R1 (5′-GTTGTTCTTAGCGAGTTGACATGTG-3′). PCR analysis for the identification of *mtsin1a-1* and *mtsin1a-2* was performed with an annealing temperature of 55 °C for 32 cycles using EasyTaq DNA Polymerase (TransGen Biotech, Beijing, China). The products of the 32-cycle PCR were examined by electrophoresis on a 1.5% (*w*/*v*) agarose gel. For RT-PCR analysis, a 1257-bp transcript fragment of *MtSIN1a* was amplified from leaf cDNA of the WT, *mtsin1a-1*, and *mtsin1a-2* with primers MtSIN1a-RT-F (5′-ATGATAATGGGAATTCAAGACAA-3′) and MtSIN1a-RT-R (5′-TTGTTGGAATCCAAATCCAA-3′), with *MtActin* (MtActin-F: 5′-ACGAGCGTTTCAGATG-3′; MtActin-R: 5′-ACCTCCGATCCAGACA-3′) serving as an control [25]. RT-PCR analyses were performed with a 55 °C annealing temperature for 28 cycles using EasyTaq DNA Polymerase (TransGen Biotech). Products of the 28-cycle PCR were examined by electrophoresis in 1.5% (*w*/*v*) agarose gel.

### 2.9. RNA Extraction and RT-qPCR Analysis

Total RNA was extracted from tissues (roots: the entire root system of the plant; stems: stem segments of the plant; adult leaves: fully expanded green leaves; flowers: fully opened flowers; pods: mature pods in the plant; flower buds: unopened flowers) using RNAiso Plus reagent (TaKaRa, Tokyo, Japan). Samples of 100 mg from different tissues were collected for RNA extraction and analysis of relative gene expression levels. Plant materials were homogenized into a fine powder using a Tissuelyser-48 (Shanghai Jingxin, Shanghai, China). About 2 μg of RNA was treated with DNase I (TransGen Biotech, Beijing, China). The DNA-free RNA was used to synthesize cDNA using 5× All-In-One RT MasterMix (Applied Biological Materials, Richmond, BC, Canada). The primers used for RT-qPCR were as follows: MtSIN1a-qRT-F (5′-ACAGCAACGGTTCATCGTCT-3′) and MtSIN1a-qRT-R (5′-AGCAACGGTCGTCAATCTGT-3′); MtSIN1b-qRT-F (5′-TTCAAAGCGGCATGAGGACT-3′) and MtSIN1b-qRT-R (5′-GGTCACCCAAGCCCGATAAA-3′); MsSOD1-qRT-F (5′-CCTGGTGGTGGTGGAAAGC-3′) and MsSOD1-qRT-R (5′-AGCGATGCCCATCCTGAAC-3′); MsSOD2-qRT-F (5′-GAGCCAGAATACGTTTGAG-3′) and MsSOD2-qRT-R (5′-TTCTAGTGACTTCCCATCT-3′). RT-qPCR was set up in a 10 μL reaction system using 2 × M5 SYBR Green Mix (Mei5bio, Beijing, China). Gene expression profiles were normalized relative to *MtUBIQUITIN or MsActin* (MtUBIQUITIN-F: 5′-CTGACAGCCCACTGAATTGTGA-3′; MtUBIQUITIN-R: 5′-TTTTGGCATTGCTGCAAGC-3′; MsActin-F: 5′-ACTCACACCGTCACCAGAATCC-3′; MsActin-R: 5′-TCAATGTGCCTGCCATGTATGT-3′) and calculated using the 2^−ΔΔCt^ method, with three independent biological replicates [26]. To investigate the responses of *MtSIN1a* and *MtSIN1b* to saline stress treatment, we used RT-qPCR to analyze their expression levels in adult leaves at 0, 3, 6, 9, 12, and 24 h after salinity treatment.

### 2.10. Plasmid Construction and Plant Transformation

The coding region of *MtSIN1a* was amplified and cloned into the pENTR/D-TOPO vector (Invitrogen, Carlsbad, CA, USA). The fusion was subsequently introduced into the destination vector *pEarleyGate* 103 through Gateway LR recombination (Thermo Fisher Scientific, Waltham, MO, USA). The primers used for *MtSIN1a* were MtSIN1a-CDS-F (5′-ATGATAATGGGAATTCAAGACAA-3′) and MtSIN1a-CDS-R (5′-TTGTTGGAATCCAAATCCAA-3′). For plant transformation *Agrobacterium tumefaciens* strain EHA105 carrying the target vector (*35S:MtSIN1a-GFP*) was used to infect SY4D leaves [27].

### 2.11. Subcellular Localization Analysis

The *A. tumefaciens* strain EHA105 harboring either *35S:MtSIN1a-GFP* or *35S:MtSIN1b-GFP* was used to infiltrate *Nicotiana tabacum* leaves. The primers used for subcellular localization analysis were as follows: MtSIN1a-CDS-F, MtSIN1a-CDS-R; MtSIN1b-CDS-F (5′-ATGGGAGTTCCAGAGAGAGATCCTCTTTC-3′), MtSIN1b-CDS-R (5′-ATGACCCGAATACCCAAACCCGAAT-3′). Additionally, *35S:GFP* was used as the control, following established protocols [28]. Fluorescence signals were observed using a Zeiss LSM 880 confocal laser scanning microscope.

### 2.12. Transcriptomic Analysis

For the transcriptomic analysis, four-week-old plants’ leaf samples were harvested. Three biological replicates were included. RNA was extracted and libraries were constructed and sequenced (BGI Genomics, Wuhan, China). The raw sequencing data were filtered with SOAPnuke to obtain clean data [29]. The RNA-seq reads were then aligned to the reference genome (Medicago_sativa_3879.figshare.v2020-07-09.v2201) using HISAT2 (Hierarchical Indexing for Spliced Alignment of Transcripts) [30]. Subsequently, the clean data were aligned to the reference gene set using Bowtie2 (v2.3.4.3) [31]. Gene expression quantification was performed with RSEM (v1.3.1) [32], and differential gene expression analysis was conducted using DESeq2 (v1.4.5) with a significance threshold of a q-value ≤ 0.05 or FDR ≤ 0.001 [33]. To further investigate the biological functions of the differentially expressed genes (DEGs) associated with the phenotypic changes, Gene Ontology (GO, http://www.geneontology.org/, accessed on 15 August 2025) and Kyoto Encyclopedia of Genes and Genomes (KEGG, https://www.kegg.jp/, accessed on 15 August 2025) enrichment analyses were performed based on the hypergeometric test using the Phyper tool. Terms with a q-value ≤ 0.05 (http://github.com/jdstorey/qvalue, accessed on 15 August 2025) were considered significantly enriched among the candidate genes.

### 2.13. Statistical Analyses

For gene expression level and plant phenotype analyses, Student’s *t*-test was employed to determine whether observed differences were statistically significant [25].

## 3. Results

### 3.1. The Expression of MtSIN1a Was Activated Under Salinity Stress Treatment

To identify GmSIN1 homologs in *M. truncatula*, we conducted a homolog search in the *M. truncatula* genome and identified two homologous genes, *MtSIN1a* and *MtSIN1b* (Figure S1).

**Figure 1:**
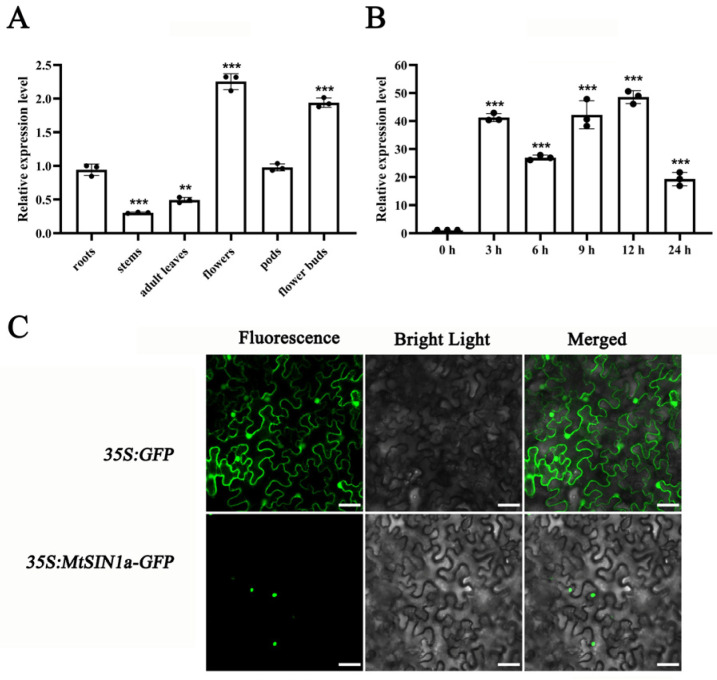
The expression pattern and response to salinity stress of *MtSIN1a*. (**A**) Relative expression levels of *MtSIN1a* in wild-type roots, stems, adult leaves, flowers, pods, and flower buds detected by RT-qPCR. Values are means ± SD of three biological replicates. Statistical significance was determined using Student’s *t*-test (** *p* < 0.01, *** *p* < 0.001; “***” indicates a significant difference in relative expression level compared to the roots). (**B**) Relative expression levels of *MtSIN1a* in WT leaves detected by RT-qPCR with NaCl treatment. Values are means ± SD of three biological replicates. Statistical significance was determined using Student’s *t*-test (*** *p* < 0.001; “***” indicates a significant difference in relative expression level compared to at 0 h). (**C**) Subcellular localization of MtSIN1a-GFP in *Nicotiana benthamiana* leaf epidermal cells. Bars = 200 μm.

To learn the roles of *MtSIN1a* and *MtSIN1b* in plant development, we investigated the expression patterns of these two genes in various tissues. The results showed that both genes were constitutively expressed in most tissues, including the roots, adult leaves, pods, flowers, and flower buds, while *MtSIN1a* expression was also detected in the stems (Figure 1A and Figure S2A). The expression of *MtSIN1a* showed constant activation during the investigated time frame, with the highest level at 12 h (a 48-fold increase compared with the 0 h sample) and a gradual decline to 18-fold up-regulation at 24 h (Figure 1B). The *MtSIN1b* gene showed a similar pattern as *MtSIN1a* with a relatively lower transcriptional activation, with the a 35-fold up-regulation at 9 h (Figure S2B).

As MtSIN1a and MtSIN1b belong to the NAC transcription factor family, we assessed their subcellular localization using the GFP fusion protein. The fluorescence microscope observation showed that both MtSIN1a-GFP and MtSIN1b-GFP were localized in nuclei (Figure 1C and Figure S2C). The expression and subcellular localization results indicate that *MtSIN1s* function as transcription regulators in salinity response in Medicago.

### 3.2. Mutants mtsin1a Displayed Enhanced Salinity Sensitivity

**Figure 2:**
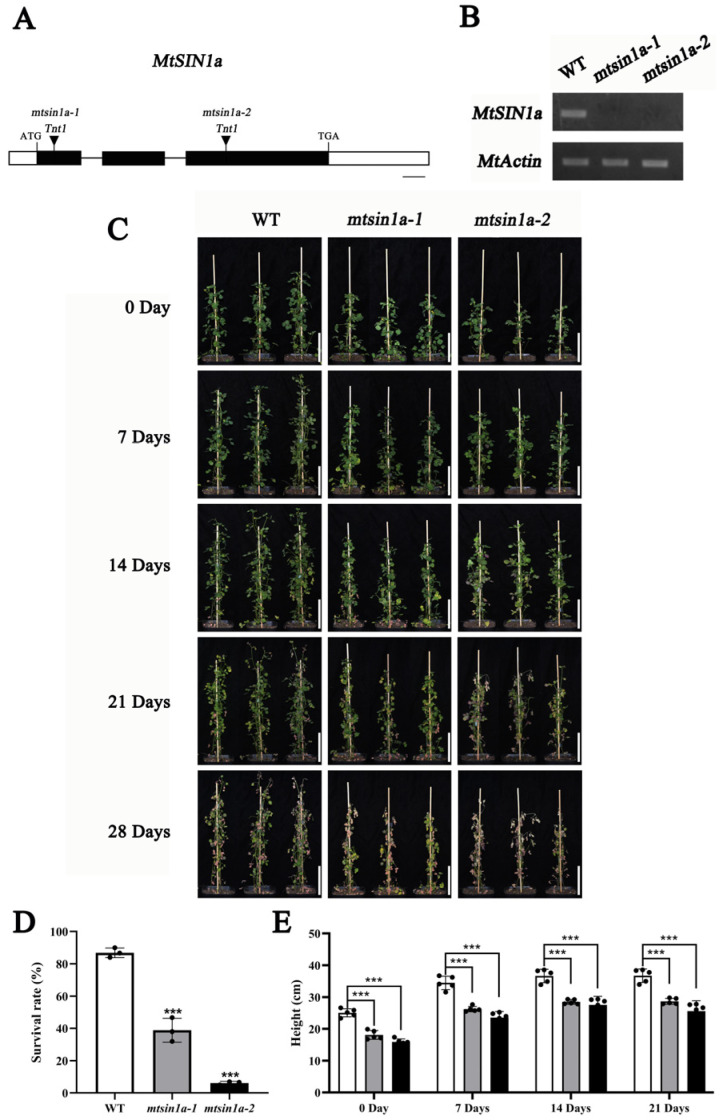
Mutants *mtsin1a* displayed enhanced salinity sensitivity. (**A**) Schematic representation of the *MtSIN1a* gene structure and *Tnt1* insertion sites. Exons are represented by boxes, introns by lines, and the *Tnt1* retrotransposon insertion positions are indicated by triangle arrows. (**B**) RT-PCR analysis of *MtSIN1a* transcripts in the WT, *mtsin1a-1*, and *mtsin1a-2*. *MtActin* was used as the control. (**C**) Phenotypic analysis of the WT, *mtsin1a-1*, and *mtsin1a-2* under 100 mM NaCl treatment at 0, 7, 14, 21, and 28 days. Bars = 10 cm. (**D**) Survival rates in the WT, *mtsin1a-1*, and *mtsin1a-2* after 28-day 100 mM NaCl treatment. Values represent the mean ± SD of three biological replicates contained in 25 plants. Statistical significance was determined using Student’s *t*-test (*** *p* < 0.001; “***” indicates that the survival rates of *mtsin1a-1* and *mtsin1a-2* at 28 days were significantly lower than that of the WT). (**E**) Plant height in the WT, *mtsin1a-1*, and *mtsin1a-2* under 100 mM NaCl treatment at 0, 7, 14, and 21 days. Values represent the mean ± SD of three biological replicates. Statistical significance was determined using Student’s *t*-test (*** *p* < 0.00; “***” indicates that the plant heights of *mtsin1a-1* and *mtsin1a-2* were significantly lower than those of the WT). White columns: WT; gray columns: *mtsin1a-1*; black columns: *mtsin1a-2*.

Because *MtSIN1a* showed constitutive expression in the shoots and stronger up-regulation during salinity treatment, we chose *MtSIN1a* for further analysis. To study the role of *MtSIN1a* in salinity response, we screened the *M. truncatula Tnt1* mutant collection and found two *mtsin1a* lines. The two *mtsin1a* lines contained *Tnt1* insertions in the first and third exon in *mtsin1a-1* and *mtsin1a-2*, respectively (Figure 2A). The *Tnt1* insertions were predicted to disrupt the *MtSIN1a* gene structure, which was verified by the presence of undetected *MtSIN1a* expression in the mutant plants (Figure 2B). In the wild-type plants, the salinity treatment caused chlorosis at 14 days after NaCl treatment, the leaves turned yellow at 21 days, and the plants showed wilting at 28 days. In the *mtsin1a* mutants, chlorosis was observed at 7 days, the leaves started to yellow at 14 days, and the plants showed wilting earlier than 21 days (Figure 2C). Following 28 days of salt stress treatment, the survival rates of *mtsin1a-1*, *mtsin1a-2*, and the WT were 46%, 6%, and 86%, respectively (Figure 2D). The plant heights of the WT during this treatment were 25.08 cm and 36.72 cm at 0 day and 21 days, while those of *mtsin1a-1* were 18.13 cm and 28.70 c and those of *mtsin1a-2* were 15.95 cm and 25.61 cm (Figure 2E). These results demonstrate that the knock-out of *MtSIN1a* impaired the salinity tolerance of *M. truncatula*.

### 3.3. The Transgenic Expression of MtSIN1a Conferred Salinity Tolerance in Alfalfa with Enhanced ROS Scavenging

**Figure 3:**
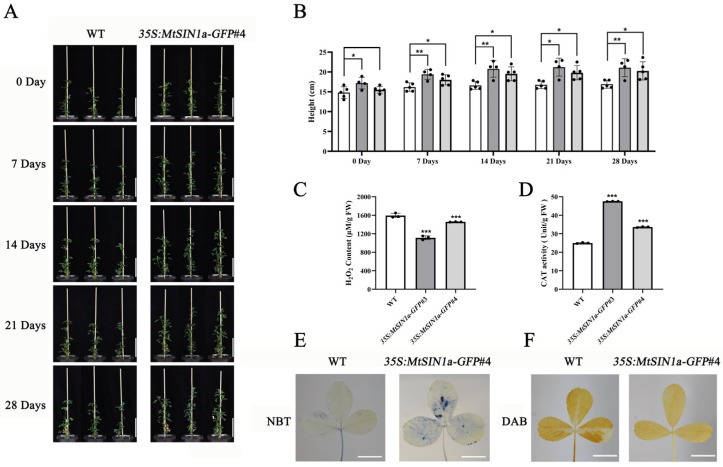
The transgenic expression of *MtSIN1a* conferred salinity tolerance in alfalfa with enhanced ROS scavenging. (**A**) Phenotypic analysis of the WT and *35S:MtSIN1a-GFP*#4 under sequential NaCl treatments. Bars = 10 cm. (**B**) Plant height in the WT, *35S:MtSIN1a-GFP*#3, and *35S:MtSIN1a-GFP*#4 under sequential NaCl treatments. Values represent the mean ± SD of three biological replicates. Statistical significance was determined using Student’s *t*-test (* *p* < 0.05, ** *p* < 0.01; “**” indicates that the plant height of *35S:MtSIN1a-GFP* was significantly higher than that of the WT). White columns: WT; dark gray columns: *35S:MtSIN1a-GFP*#3; light gray columns: *35S:MtSIN1a-GFP*#4. (**C**) H_2_O_2_ content in the WT, *35S:MtSIN1a-GFP*#3, and *35S:MtSIN1a-GFP*#4. Values represent the mean ± SD of three biological replicates. Statistical significance was determined using Student’s *t*-test (*** *p* < 0.001; “***” indicates that H_2_O_2_ content in the leaves of *35S:MtSIN1a-GFP* was significantly lower than that in the WT). (**D**) CAT enzyme activity in the WT, *35S:MtSIN1a-GFP*#3, and *35S:MtSIN1a-GFP*#4. Values represent the mean ± SD of three biological replicates. Statistical significance was determined using Student’s *t*-test (*** *p* < 0.001; “***” indicates that CAT enzyme activity in the leaves of *35S:MtSIN1a-GFP* was significantly higher than that in the WT). (**E**) NBT staining of the WT and *35S:MtSIN1a-GFP*#4 leaves. Bars = 1 cm. (**F**) DAB staining of the WT and *35S:MtSIN1a-GFP*#4 leaves. Bars = 1 cm.

The discoveries reported above raised questions regarding the role of *MtSIN1a* in alfalfa under salinity stress. To answer the question of the role of *MtSIN1a*, we expressed *MtSIN1a* in alfalfa by transforming the construct of *35S:MtSIN1a-GFP* in the tetraploid SY4D line. Among the seven transgenic lines of *35S: MtSIN1a-GFP* alfalfa plants we obtained, we chose the transgenic lines *35S:MtSIN1a-GFP*#3 and *35S:MtSIN1a-GFP*#4, with high expression levels of *MtSIN1a* (Figure S3), for subsequent studies. The alfalfa plants of the WT and *35S:MtSIN1a-GFP* that had developed at the same stage with comparable plant heights were used in a four-week NaCl treatment (100, 150, 200, and 250 mM, sequentially). The *35S:MtSIN1a-GFP* plants showed higher plant heights compared with the wild type at each stage of the treatment (Figure 3A,B). These results demonstrate that the transgenic alfalfa plants expressing *MtSIN1a* showed enhanced tolerance to salinity stress.

To understand the mechanism underlying transgenic alfalfa’s salinity tolerance, we assessed the ROS content in *35S:MtSIN1a-GFP* plants as well as in the WT. The content of H_2_O_2_ was measured and we found it to be reduced in *35S:MtSIN1a-GFP* plants compared with the WT (Figure 3C), which was consistent with a weaker DAB staining in the transgenic plants (Figure 3F). The signals of NBT were found to be increased in *35S:MtSIN1a-GFP*#4 leaves, indicating an elevated O_2_^−^ content (Figure 3E). The transgenic and WT plants were also used for MDA assessment after NaCl treatment, and the results showed a reduced MDA content and therefore alleviated ROS damage in the *35S:MtSIN1a-GFP* plants (Figure S4). In the *35S:MtSIN1a-GFP* plants, we detected an increase in the activity of the ROS-scavenging CAT, which indicates that the ROS scavenging was activated in transgenic plants expressing *MtSIN1a* (Figure 3D).

### 3.4. The Ribosome and ROS-Scavenging Pathways Were Activated in 35S:MtSIN1a Transgenic Alfalfa

To study the mechanisms underlying *35S:MtSIN1a* transgenic alfalfa’s tolerance to salinity, we carried out a transcriptomic analysis of non-treated plants. By comparing WT and *35S:MtSIN1a*#4 transgenic plants, we identified 170 differentially expressed genes (DEGs) with over two-fold changes in expression and a q-value smaller than 0.05, including 125 up-regulated and 45 down-regulated DEGs (Supplementary Tables S2–S5, Figure S5).

**Figure 4:**
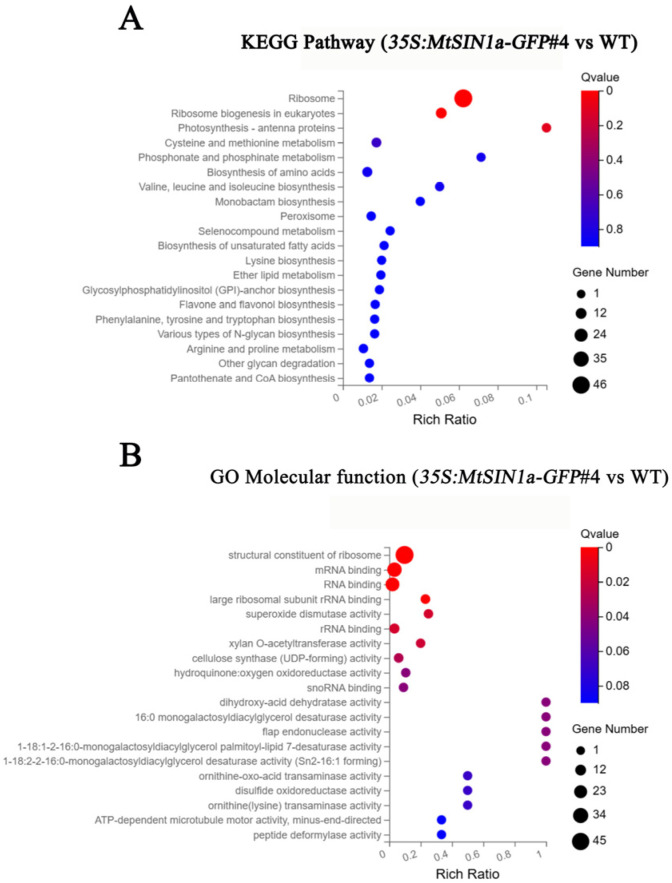
The ribosome and ROS scavenging pathways were activated in *35S:MtSIN1a* transgenic alfalfa. (**A**) KEGG pathway analysis of differentially expressed genes in *35S:MtSIN1a-GFP*#4 compared with the WT; (**B**) GO Molecular Function analysis of differentially expressed genes in *35S:MtSIN1a-GFP*#4 compared with the WT.

The Kyoto Encyclopedia of Genes and Genomes (KEGG) enrichment analysis revealed that the ribosome, ribosome biogenesis, photosynthesis-antenna protein, and photosynthesis pathways were significantly enriched in the DEGs (q-value < 0.05, Figure 4A). In addition, 55 genes in the ribosome and ribosome biogenesis pathways were up-regulated, and 2 genes in the photosynthesis and antenna protein pathways were down-regulated. The most differentially regulated DEGs included 46 genes involved in the ribosome (Figure 4A). Interestingly, the *35S:MtSIN1a-GFP*#4 transgenic plants exhibited higher dry weights at the harvest stage (Figure S6), indicating that the protein synthesis was probably enhanced by the ribosome biogenesis pathway involved in the nucleoli’s shape, size, and number.

**Figure 5:**
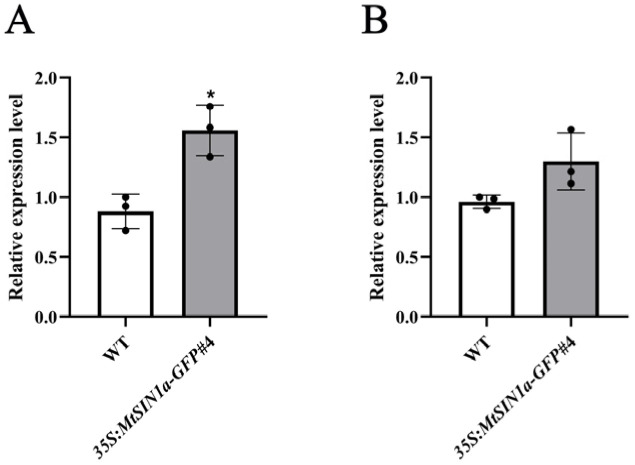
The expression level of *MtSODs* was enhanced in *35S:MtSIN1a* transgenic alfalfa. (**A**) Relative expression levels of *MsSOD1* in the WT and *35S:MtSIN1a-GFP*#4. Values represent the mean ± SD of three biological replicates; statistical significance was determined using Student’s *t*-test (* *p* < 0.05; indicating that the relative expression level of *MsSOD1* in the *35S:MtSIN1a-GFP*#4 line was significantly higher than that in the WT under untreated conditions). (**B**) Relative expression levels of *MsSOD2* in the WT and *35S:MtSIN1a-GFP*#4. Values represent the mean ± SD of three biological replicates; statistical significance was determined using Student’s *t*-test.

The Gene Ontology (GO) enrichment analysis revealed that genes encoding structural constituents of the ribosome, mRNA binding, RNA binding, large ribosomal subunit rRNA binding, superoxide dismutase activity, rRNA binding, xylan O-acetyltransferase activity, cellulose synthase (UDP-forming) activity, hydroquinone, oxygen oxidoreductase activity, snoRNA binding, dihydroxy-acid dehydratase activity, and flap endonuclease activity were significantly enriched in the DEGs (q-value < 0.05, Figure 4B). Among the GO enrichment analysis results above, the genes encoding superoxide dismutase activity were involved in ROS scavenging, with *MsG0180002545* and *MsG0180002098* encoding *MsSOD1* and *MsSOD2*. The RT-qPCR results showed that the expression of *MsSOD1* was activated in *35S:MtSIN1a* transgenic plants (Figure 5A). These results show that the ribosome and ROS scavenging were activated, indicating that tolerance was enhanced by ROS scavenging in transgenic plants expressing *MtSIN1a*.

## 4. Discussion

In this study, we characterized two *MtSIN1s* in Medicago during salinity treatment. We observed their salinity-responding activation and found that *mtsin1a* knock-out *M. truncatula* plants displayed enhanced susceptibility to salinity. When expressing *MtSIN1a* in alfalfa, the transgenic plants showed enhanced salinity tolerance with an elevated ROS scavenging pathway. This study demonstrated the role of *MtSIN1a*’s role in salinity tolerance, which can guide future alfalfa breeding for enhanced salt tolerance.

High levels of salinity led to cell damage in the plants. Research on these plants’ responses to saline stress has uncovered pathways including the production and scavenging of ROS, such as O_2_^−^ and H_2_O_2_ [34]. On the one hand, ROS are synthesized by Rbohs. On the other hand, ROS are scavenged step by step. O_2_^−^ is catalyzed to H_2_O_2_ by the action of SOD. H_2_O_2_ is then catalyzed to water (H_2_O) by the action of CAT and APX. Though ROS can activate the signaling pathway to respond to abiotic stress, an excess amount of ROS can cause cell damage [35]. ROS-scavenging enzymes, SOD and CAT, are then activated to remove the extra ROS. The expression levels of *MsSODs* showed activation in *35S:MtSIN1a-GFP*#4 transgenic alfalfa (Figure 5), indicating that the ROS homeostasis between production and scavenging was altered.

Interestingly, the ROS pathway was also found to be altered in *GmSIN1*-overexpressing transgenic soybeans in a previous study [10]. The similarity between *GmSIN1* and *MtSIN1a* is seen in the stronger NBT staining signals, indicating the similar roles in O_2_^−^ of these two genes. The difference between these two studies is found in the DAB staining signals. In our study, the *35S:MtSIN1a-GFP* transgenic alfalfa contained less H_2_O_2_ (Figure 3), indicating that the regulatory network of *MtSIN1a* was different from that of *GmSIN1* in H_2_O_2_ production and scavenging. A high content of ROS can cause plant development arrestment. The height of the WT and transgenic plants were compared and the results showed that *MtSIN1a* conferred enhanced plant heights (Figure 3), indicating the positive role of *MtSIN1a* in ROS homeostasis between development and salinity tolerance.

Last but not least, not only was the height enhanced, but the dry weight of the *35S:MtSIN1a-GFP*#4 alfalfa was also elevated (Figure S6). The transcriptomic analysis showed that the ribosome and ribosome biogenesis pathways were both enhanced in the *35S:MtSIN1a-GFP* alfalfa according to the KEGG (Figure 4). There were 55 genes belonging to the ribosome and ribosome biogenesis pathways in the 125 up-regulated DEGs (Supplementary Tables S4 and S5). The ribosome and ribosome biogenesis pathways were found to play important roles in protein processing, translational fidelity, and nucleoli-associated protein synthesis [36]. The enhanced plant height and dry weight with the elevated ribosome and ribosome biogenesis pathways indicated that more protein was synthesized with a high quality in the transgenic plants at the harvest time. These results suggest that *MtSIN1a* enhanced the plants’ salinity tolerance, with activated ribosome and ribosome biogenesis pathways contributing to protein metabolism.

Overall, we describe *MtSIN1a*’s role in regulating salinity tolerance, finding that the tolerance to saline stress can be enhanced by the activation of ROS scavenging in alfalfa. This study provides useful genes for the molecular breeding of alfalfa with enhanced salinity stress tolerance. Therefore, our work showcases a novel way to characterize candidate genes from leguminous relatives in diploid Medicago, and assess their utilization in the tetraploid alfalfa.

## Data Availability

Datasets generated during this study are available at SRA with the BioSample accession number PRJNA1311504.

## Supplementary Materials

The following supporting information can be downloaded at https://www.mdpi.com/article/10.3390/genes16101156/s1. Figure S1: Phylogenetic analysis of MtSIN1a; Figure S2: The expression pattern and response to salinity stress of *MtSIN1b*; Figure S3: The Relative expression levels of *MtSIN1a* in *35S:MtSIN1a* transgenic alfalfa; Figure S4: MDA content in *35S:MtSIN1a* transgenic alfalfa; Figure S5: Differentially expressed genes between *35S:MtSIN1a-GFP*#4 and WT; Figure S6: Dry weight in WT and *35S:MtSIN1a-GFP*#4 after NaCl treatment; Table S1: The homologs of GmSIN1; Table S2: 125 up-regulated DEGs in *35S:MtSIN1a*#4 compared to WT; Table S3: 45 down-regulated DEGs in *35S:MtSIN1a*#4 compared to WT; Table S4: Identification of ribosome genes among up-regulated DEGs; Table S5: Identification of ribosome biogenesis genes among up-regulated DEGs.
