# Supplementary material for: MtSIN1a Enhances Salinity Tolerance in Medicago truncatula and Alfalfa
Source: Genes (Basel). 2025 Sep 29;16(10):1156. doi: 10.3390/genes16101156 (PMC12564375; doi:10.3390/genes16101156)
Supplement: Supplementary file 1 [file genes-16-01156-s001.zip › Figure S Legend.pdf]

Figure S1
Phylogenetic analysis of MtSIN1a.

Figure S2 The expression pattern and response to salinity stress of *MtSIN1b*.
(A) Relative expression levels of *MtSIN1b* in wild-type roots, stems, adult leaves, flowers, pods, and flower buds were detected by RT-qPCR. Values are means ± SD of three biological replicates. Statistical significance was determined by the Student's *t*-test. (*P < 0.05, **P < 0.01, ***P < 0.001, "***" represents a significant difference in relative expression level compared to the roots)
(B) Relative expression levels of *MtSIN1b* in wild-type leaves were detected by RT-qPCR within 0-24 h of 100 mM NaCl treatment. Values are means ± SD of three biological replicates. Statistical significance was determined by the Student's *t*-test. (*P < 0.05, **P < 0.01, ***P < 0.001, "***" represents a significant difference in relative expression level compared to the 0 h)
(C) Subcellular localization of MtSIN1b-GFP in *Nicotiana benthamiana* leaf epidermal cells. Bars = 200 μm.

Figure S3 The Relative expression levels of *MtSIN1a* in *35S:MtSIN1a* transgenic alfalfa.
Identification of the relative expression level of *MtSIN1a* in *35S:MtSIN1a-GFP* transgenic plants. Values represent the mean ± SD of three biological replicates; Statistical significance was determined by the Student's *t*-test (*P < 0.05, **P < 0.01, ***P < 0.001, "***" represents that the relative expression level of *MtSIN1a* in *35S:MtSIN1a-GFP* was significantly higher than that of WT).

Figure S4 MDA content in *35S:MtSIN1a* transgenic alfalfa.
MDA content in WT, *35S:MtSIN1a-GFP*#3 and *35S:MtSIN1a-GFP*#4 plants before and after salt treatment (14 days). Values represent the mean ± SD of three biological replicates; Statistical significance was determined by the Student's *t*-test (*P < 0.05, **P < 0.01, ***P < 0.001, "***" represents that MDA content of *35S:MtSIN1a-GFP*#3 before salt treatment were significantly lower than that of WT, "***" represents that MDA content of *35S:MtSIN1a-GFP*#4 after salt treatment (14 days) were significantly lower than that of WT).

Figure S5 Differentially expressed genes between *35S:MtSIN1a-GFP*#4 and WT.

Figure S6 Dry weight in WT and *35S:MtSIN1a-GFP*#4 after NaCl treatment.
Dry weight in WT and *35S:MtSIN1a-GFP*#4 after 28-day NaCl treatment. Values represent the mean ± SD of three biological replicates; Statistical significance was determined by the Student's *t*-test (*P < 0.05, "*" represents that dry weight of *35S:MtSIN1a-GFP*#4 lines at 28 days was significantly heavier than that of WT).
