# Supplementary figures and images for: MtSIN1a Enhances Salinity Tolerance in Medicago truncatula and Alfalfa
Source: Genes (Basel). 2025 Sep 29;16(10):1156. doi: 10.3390/genes16101156 (PMC12564375; doi:10.3390/genes16101156)

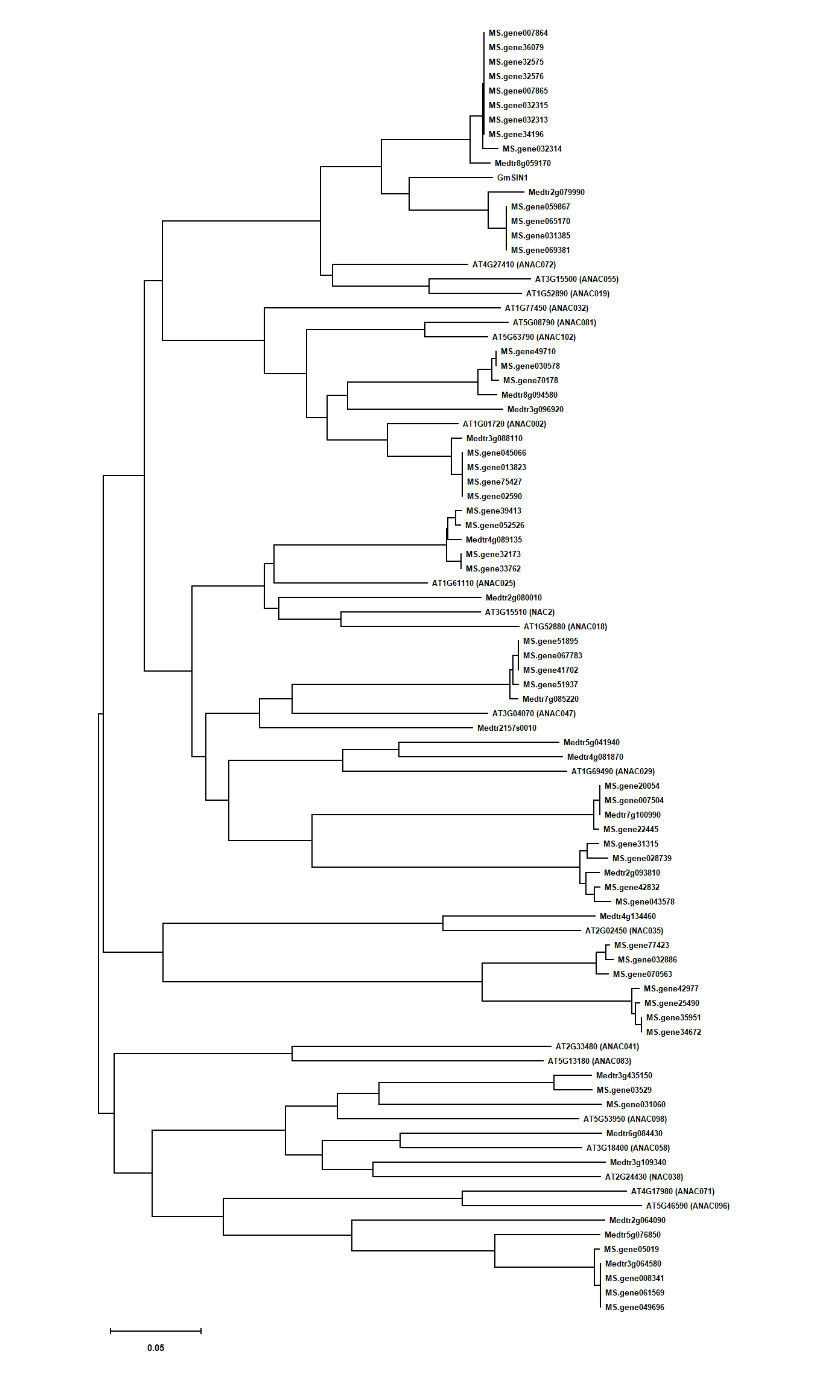

Supplement: Supplementary file 1 [file genes-16-01156-s001.zip › Figure S1.tif]

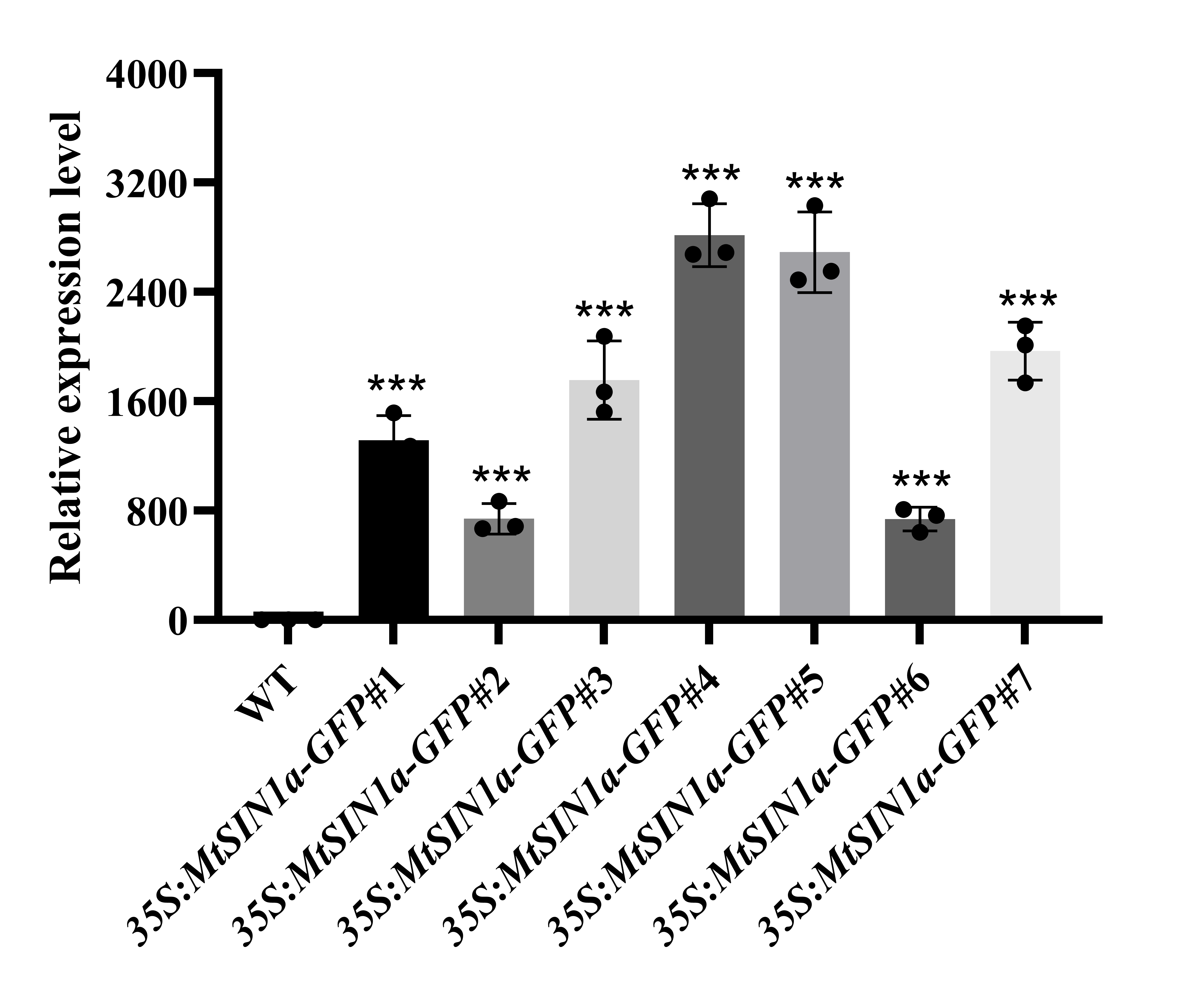

Supplement: Supplementary file 1 [file genes-16-01156-s001.zip › Figure S3.tif]

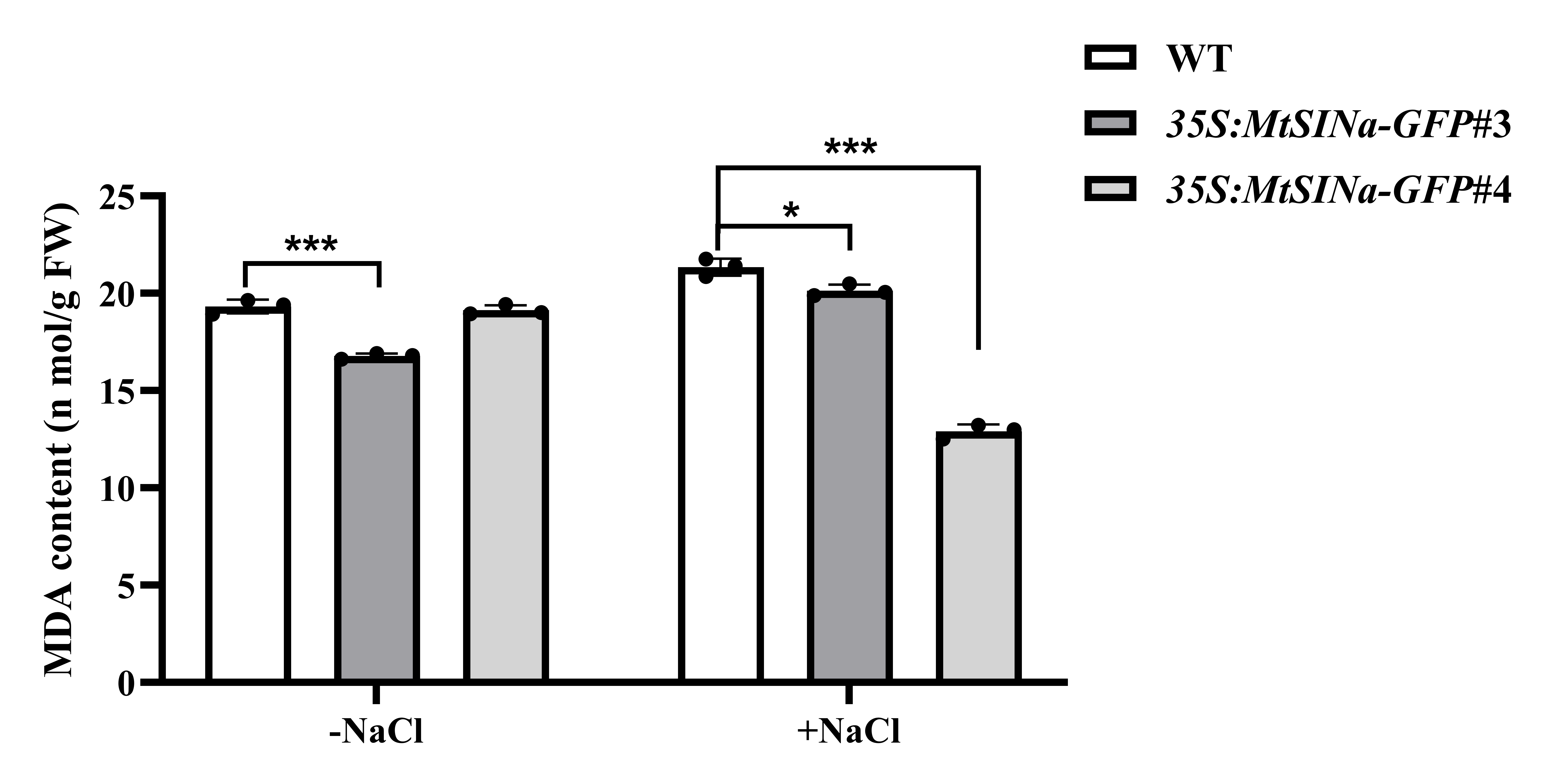

Supplement: Supplementary file 1 [file genes-16-01156-s001.zip › Figure S4.tif]

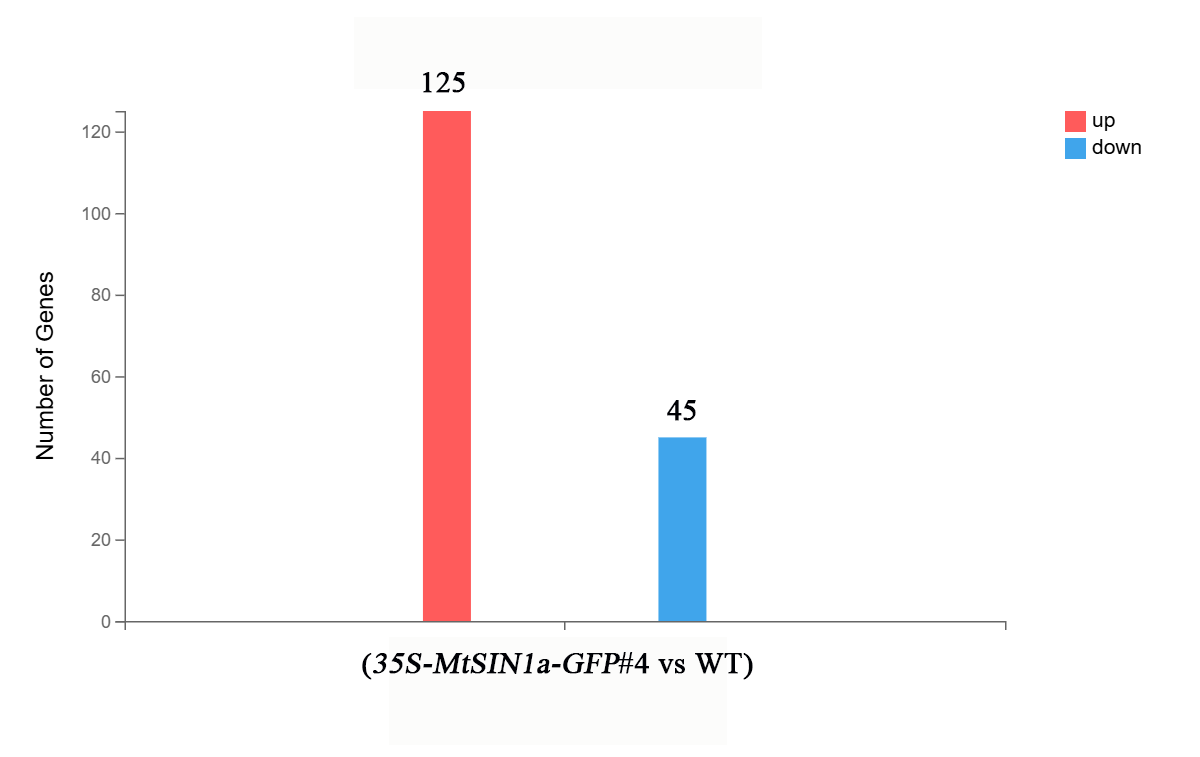

Supplement: Supplementary file 1 [file genes-16-01156-s001.zip › Figure S5.tif]

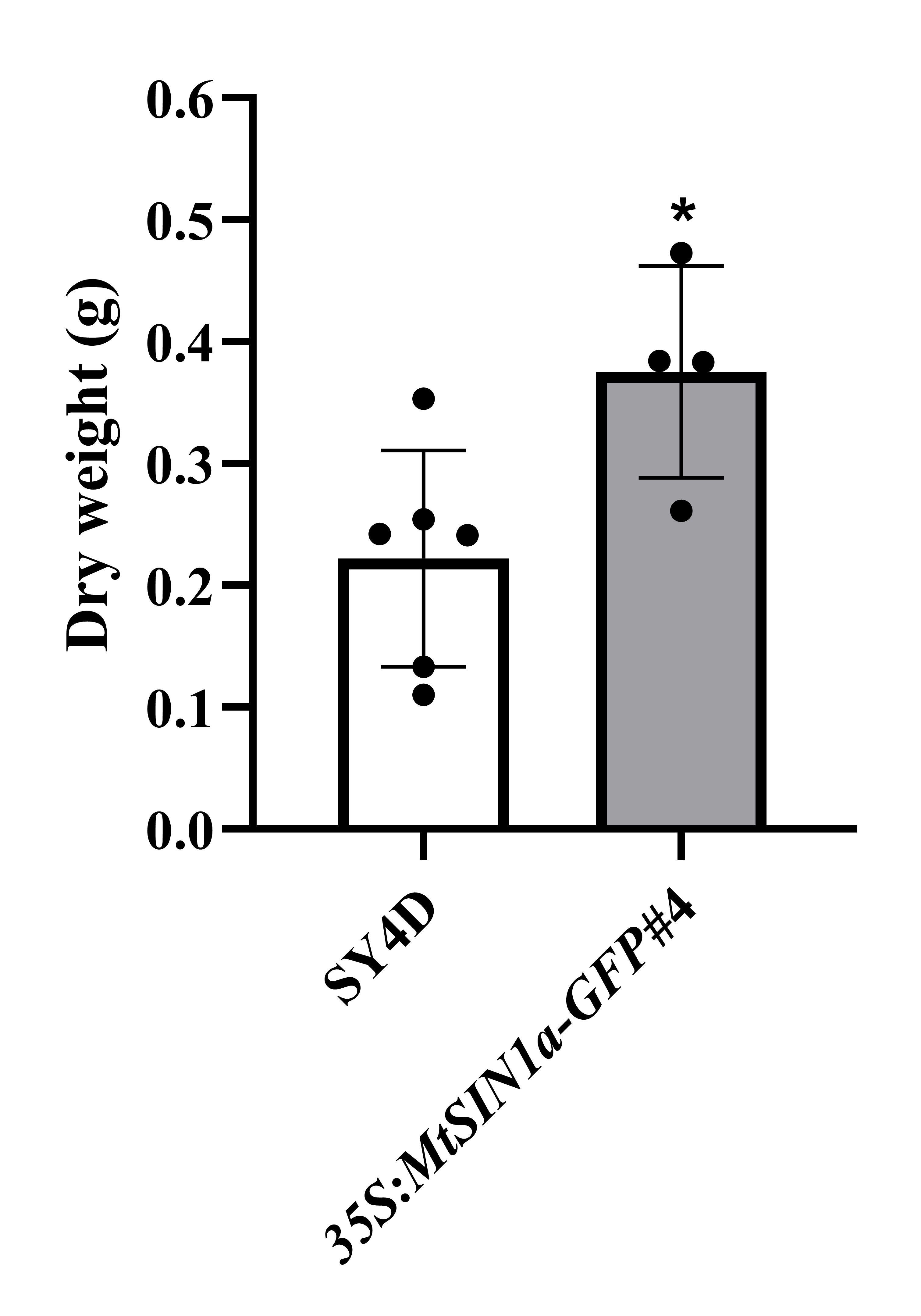

Supplement: Supplementary file 1 [file genes-16-01156-s001.zip › Figure S6.tif]
